# Biochemistry: one molecule at a time

**DOI:** 10.1042/EBC20210015

**Published:** 2021-04-16

**Authors:** Dominika T. Gruszka

**Affiliations:** Single Molecule Imaging of Genome Duplication and Maintenance Laboratory, The Francis Crick Institute, 1 Midland Road, London NW1 1AT, U.K.

**Keywords:** biophysics, single-molecule

## Abstract

Biological processes are orchestrated by complex networks of molecules. Conventional approaches for studying the action of biomolecules operate on a population level, averaging out any inhomogeneities within the ensemble. Investigating one biological macromolecule at a time allows researchers to directly probe individual behaviours, and thus characterise the intrinsic molecular heterogeneity of the system. Single-molecule methods have unravelled unexpected modes of action for many seemingly well-characterised biomolecules and often proved instrumental in understanding the intricate mechanistic basis of biological processes. This collection of reviews aims to showcase how single-molecule techniques can be used to address important biological questions and to inspire biochemists to ‘zoom in’ to the population and probe individual molecular behaviours, beyond the ensemble average. Furthermore, this issue of *Essays in Biochemistry* is the very first written and edited entirely by early career researchers, and so it also highlights the strength, diversity and excellence of the younger generation single-molecule scientists who drive this exciting field of research forward.

Macromolecules and their biochemical reactions govern subtle and mechanistically complex processes that are fundamental to life; e.g. protein folding, cargo trafficking, DNA replication, transcription and translation. Classic biochemical studies describe the behaviour of a large ensemble of macromolecules, and thus report the mean value of the measured parameter, averaged over the entire molecular population. Ensemble-based approaches cannot distinguish molecules with different properties within the population (e.g. different conformations) nor can they reveal how an individual molecule behaves over time. By contrast, single-molecule approaches allow us to investigate biological features ‘one molecule at a time’ and directly monitor such heterogeneous behaviours. Single-molecule measurements report the probabilistic distribution of values for a measured parameter, and so characterise not only the mean behaviour but also the likelihood of fluctuations about the mean. These fluctuations provide a direct access to the molecular heterogeneity of a biological system, which can be of static or dynamic origin. Static heterogeneity is observed when an ensemble of molecules contains subpopulations that do not change over the observation timescale whereas dynamic heterogeneity is characteristic of molecules that interconvert over the observation timescale. Hence, single-molecule studies provide insight into the dynamics, kinetics and mechanisms of biomolecular processes that are unattainable by traditional ensemble approaches.

Since the development of the first single-molecule technique, i.e. single-ion channel recordings using patch-clamp [1], the single-molecule toolkit has expanded considerably and continues to evolve. Single-molecule detection and manipulation techniques can broadly be divided into the following categories: (i) *light microscopy approaches*, including fluorescence imaging and spectroscopy [2–7], and interferometric scattering [8], (ii) *electrical conductance measurements*, including patch-clamp and nanopore-based detection [9,10], and (iii) *force-based approaches*, including optical [11] and magnetic tweezers [12], and atomic force microscopy [13]. Different single-molecule methods have different capabilities, applications and throughput, and offer different time and spatial resolutions. For example, super-resolution fluorescence microscopy can achieve localisation precision of a few tens of nanometres, breaking the diffraction limit of optical microscopy, whereas atomic force microscopy reaches atomic level spatial precision (Ångström resolution) [14,15]. In addition to the three basic groups of single-molecule techniques listed above, approaches that integrate different modalities, such as force and fluorescence-based detection, have also been described [12,16–18] and continue to be developed. These hybrid approaches enable new multiplexing possibilities, which cannot be accessed through individual methods alone. Moreover, theoretical tools utilising intensive computational analysis, such as molecular simulations [5] and modelling [10], add further insight into our understanding of single-molecule biophysics.

As the temporal and spatial resolutions of single-molecule methods have been improving over the years, the complexity of analysed samples has also increased, opening up new avenues for scientific discovery. Single-molecule studies within cells [7] or cell extracts [3] are now routinely conducted, in addition to *in vitro* assays using purified components. The *in vivo* single molecule approaches mostly involve fluorescence detection [19–23] however force-based technologies are also being developed [24,25]. Indeed, recent advances in super-resolution techniques have transformed single-molecule imaging in living cells [19–23]. By their very nature, single-molecule methods are not typically high throughput, which can present a major challenge when a large number of observations are needed to adequately characterise the system’s diversity. Many recent advances (e.g. DNA curtain technology [26]) have addressed this issue, allowing the recording of large volumes of data in single experiments and automation of data collection and downstream analysis [27].

Implementation of single-molecule approaches to study a biological system is not an easy task as it requires overcoming a variety of challenges. The difficulties are associated not only with bespoke instrumentation and sophisticated data analysis of often noisy signals but also chemical modification of macromolecules and target-specific surface immobilisation. Some of these aspects have been aided by commercially available single-molecule instruments, data processing and analysis software, as well as more efficient and user-friendly protein engineering and labelling technologies. Nevertheless, to carry out a successful single-molecule experiment one needs a broad range of skills, which go far beyond the classic biochemist’s toolkit and typically involve basic engineering, knowledge of surface and protein chemistry, operation of optical instruments, complex image and data analysis, and computer programming. For research students and postdoctoral researchers, the use of single-molecule approaches as part of their scientific journey provides an exciting and rigorous training in multiple fields at the interface of life and physical sciences.

Our understanding of biochemistry has been largely gained through investigating ensembles of macromolecules. The single-molecule approaches enable us to ask and answer entirely new types of questions, which truly probe the complexity of biological systems. The goal of this issue of *Essays in Biochemistry*, focused on *Biochemistry: One Molecule at a Time* is three-fold. First, it showcases different types of single-molecule techniques and their applications to investigate processes that are fundamental to life. Second, it aims to encourage and enthuse junior biochemists to embark on the journey of single molecules in their scientific training, as it is an exciting area of truly interdisciplinary research at the forefront of modern molecular bioscience. Third, this issue emphasises the excellence and maturity of the early career single-molecule bioscientists (Ph.D. students, postdoctoral researchers and junior principal investigators), who wrote and edited this collection of essays and who are currently helping to develop this exciting field of research. I hope that this pioneering, early career researcher focused issue of *Essays in Biochemistry* will pave the way for other initiatives celebrating the contribution and importance of junior molecular bioscientists.

## Open Access

Open access for this article was enabled by the participation of The Francis Crick Institute in an all-inclusive *Read & Publish* pilot with Portland Press and the Biochemical Society under a transformative agreement with JISC.
